# Energy-Based Medical Devices in Plastic Surgery: A Comparison of FDA-Approved Indications to Advertised Claims

**DOI:** 10.1093/asjof/ojae083

**Published:** 2024-10-01

**Authors:** Diego A Gomez, Isra Abdulwadood, Aaron Bogan, Bryn E Morris, Jeffrey M Kenkel, Robert W Bernard, Edward M Reece

## Abstract

**Background:**

Lasers and energy-based devices are commonly employed in aesthetic medicine. In the United States, the FDA regulates lasers as medical devices, restricting marketing to approved indications and making off-label claims illegal. Despite this, no comprehensive analysis of off-label marketing prevalence exists.

**Objectives:**

The authors of this study aim to compare the FDA-approved indications for 2 popular aesthetic lasers to their online advertising claims. Additionally, they seek to educate aesthetic providers on the current regulatory restrictions surrounding off-label advertising.

**Methods:**

FDA-approved indications for 2 lasers—helium plasma dermal resurfacing and 2940 nm fractional erbium-doped yttrium aluminum garnet—were obtained from the publicly available Establishment Registration & Device Listings Database. Online advertisements regarding the capabilities of each laser were collected from practice websites in the United States.

**Results:**

Our analysis of 100 online claims for each laser revealed that more than half of the websites advertising helium plasma (*n* = 59) and 44 websites advertising fractional lasers made at least 1 off-label claim. Both plastic surgeons and nonplastic surgeons made at least 1 off-label claim, with no statistically significant difference between the groups.

**Conclusions:**

Despite FDA regulation of medical devices, online advertising regarding the indications and capabilities of popular medical lasers varies widely. Patients who seek information regarding aesthetic laser treatments may encounter inaccurate and differing claims for these treatments, potentially leading to false expectations and poor patient outcomes.

**Level of Evidence: 4 (Risk):**

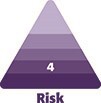

The utilization of medical lasers in aesthetic medicine has witnessed a remarkable surge over recent decades. In 2020, nearly 1 million laser skin resurfacing procedures were performed by board-certified plastic surgeons in the United States, representing a 483% increase since 2000.^1^ Online public interest has demonstrated similar exponential growth over the past decade.^2,3^ As these technologies continue to grow in popularity, it is imperative to examine the consistency between FDA-approved medical indications and the claims found in online advertisements.

Lasers and energy-based devices are employed in aesthetic medicine for a variety of purposes, including dermal resurfacing, the treatment of benign pigmented lesions, vascular abnormalities such as telangiectasias, and removal of hair and tattoos.^4^ Different laser technologies exist and can be broadly classified as ablative, nonablative (in both fractionated and unfractionated forms), or radiofrequency technologies.^5^ Ablative lasers evaporate the epidermal layer by quickly superheating tissue. Common ablative technologies include carbon dioxide (CO_2_) and erbium-doped yttrium aluminum garnet (Er:YAG). Nonablative lasers produce more mild results by causing controlled dermal injury and stimulating dermal remodeling and collagen production. Fractionation creates a regular pattern of columns of tissue injury and is available in both ablative and nonablative forms.

This review focuses on 2 popular energy-based devices: the 2940 nm Fractional Er:YAG and helium plasma dermal resurfacing (HPDR). Er:YAG lasers were first approved by the FDA in 1996 for skin resurfacing.^6^ The 2940 nm fractional Er:YAG laser wavelength has the highest absorption coefficient for water among all ablative laser wavelengths.^7^ The laser is capable of skin resurfacing and stimulating collagen production by vaporizing the epidermal layer and causing thermal injury in the dermis. Plasma results from the interaction of radiofrequency with a gas, such as helium, nitrogen, or argon. HPDR employs helium gas, which is passed over an energized electrode to enable propagation and delivery of radiofrequency to the tissues, resulting in thermal energy that produces protein denaturation and soft tissue coagulation.^8^ These devices were first approved for subdermal tissue heating to reduce skin laxity in all skin types, and are now FDA cleared for facial skin resurfacing in Fitzpatrick skin Type I, II, or III.^8^

In the United States, aesthetic lasers are federally regulated as medical devices by the FDA. A company that intends to market a medical laser must first receive approval from the FDA before commercial distribution. Each device may only be marketed under the specific indications for which it has been approved. Off-label use of a medical device is legal, provided that its use is based on firm scientific rationale. However, misbranding, which involves promoting or advertising the use of a device for anything other than its FDA-approved use, is illegal.^9^ The policy prohibiting physicians from marketing or advertising off-label uses to the general public is outlined in a 2000 letter addressed to 2 New York State physicians.^10^ These physicians had promoted the off-label use of silicone oil (AdatoSil 5000, Chiron Vision Corp., a division of Novartis Vaccines and Diagnostics, Inc., Emeryville, CA) for treating facial lines and wrinkles in a patient-directed flyer. In their letter, the US FDA stated that this advertising violated the Federal Food, Drug, and Cosmetic Act, emphasizing that a “licensed practitioner may not promote a medical device for use(s) for which they have not received FDA clearance.”^10^ Despite regulations prohibiting off-label marketing of medical lasers, no analysis of the prevalence of off-label promotion exists. This study seeks to compare the FDA-approved indications for 2 popular aesthetic lasers to online advertising claims regarding their benefits. Additionally, it seeks to educate aesthetic providers on the current regulatory restrictions surrounding off-label advertising in hopes of decreasing legal risks associated with practicing aesthetic laser medicine. To our knowledge, this is the first study to identify off-label advertising claims for these devices.

## METHODS

FDA-approved indications for the 2 lasers—HPDR and 2940 nm fractional Er:YAG—were obtained from the publicly available Establishment Registration & Device Listings Database on January 1, 2024.^11^ Google searches without location-based results were conducted for “2940-nm fractional Er:YAG” and “Helium plasma dermal resurfacing.” The first 100 practice sites that cited treatment options for each of these lasers were included in the study. Noncommercial websites, research articles, newsletters, and non-US sites were excluded from the study. The following data were extracted from each included website: advertised FDA-approved indications of use, off-label promotions of use, and provider specialty. Two authors (D.A.G. and I.A.) independently extracted data, whereas a third author (R.W.B.) resolved any discrepancies.

Analyses were performed using R version 4.0.3 (R Foundation for Statistical Computing) and SAS version 9.3 (SAS Institute Inc., Cary, NC).^12,13^ Descriptive analysis was performed using standard statistical procedures. The χ^2^ testing for homogeneity was utilized for comparison between categorical variables and Cochran–Armitage trend tests for comparison of ordinal variables. Statistical significance was set at *P* ≤ .05.

## RESULTS

A total of 200 websites were included in our sample. The majority (*n* = 135; 68.0%) were dedicated to surgeons, with plastic surgeons being the most common (*n* = 96; 47.5%), followed by dermatologists (*n* = 17), ear nose and throat (*n* = 16), and family doctors (*n* = 15). Table 1 lists the specialties represented in the study. We assessed these websites based on the approved indications set forth below.

**Table 1.** Advertising Provider by Specialty (*n* = 200)

Plastic surgery	96
Dermatology	17
Family medicine	15
Ear, nose, and throat	16
Obstetrics and gynecology	9
Internal medicine	5
Nurse practitioner	2
General surgery	8
Anesthesiology	4
Dentistry	3
Pediatrics	1
Esthetician	7
Ophthalmology	5
Radiology	1
Vascular surgery	1
Emergency medicine	3
Preventative medicine	1
Physician assistant	1
Chiropractor	1
Allergy and immunology	1
Nurse	2
Naturopath	1

The 2940 nm fractional Er:YAG is an ablative fractionated laser that is approved for use in soft tissue for skin resurfacing, treatment of wrinkles, epidermal nevi, telangiectasias, spider veins, actinic cheilitis, keloids, verrucae, skin tags, anal tags, keratoses, and scar revision (including acne scars).^14^ It is also indicated for use in skin resurfacing and treatment of rhytides, furrows, fine lines, textural irregularities, benign pigmented lesions, and vascular dyschromia.

HPDR is approved for the coagulation of subcutaneous soft tissues following liposuction for aesthetic body contouring, as well as to improve the appearance of lax (loose) skin in the neck and submental region. HPDR is also intended for the delivery of radiofrequency energy and/or helium plasma for cutting, coagulation, and ablation of soft tissue during open surgical procedures, and to areas where coagulation or contraction of soft tissue is required.^15^ Lastly, HPDR is approved for the treatment of moderate to severe wrinkles and rhytids, limited to patients with Fitzpatrick skin Type I, II or III.^16^

**Figure 1.**
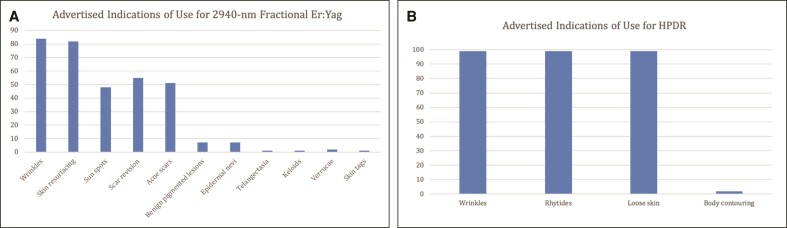
(A) Advertised FDA indications of use for 2940 nm fractional erbium-doped yttrium aluminum garnet. (B) Advertised FDA indications of use for helium plasma dermal resurfacing. HPDR, helium plasma dermal resurfacing.

All websites advertised at least 1 FDA-approved indication for use. The most frequent advertised indications of use for the 2940 nm fractional Er:YAG included wrinkles (84%), skin resurfacing (82%), scar revision (55%), acne scarring (51%), and sun spots (48%; Figure 1A). The most frequently advertised indications of use for HPDR were wrinkles (99%), rhytides (99%), and loose skin (99%), whereas only 2 websites advertised its use for body contouring (Figure 1B).

**Figure 2.**
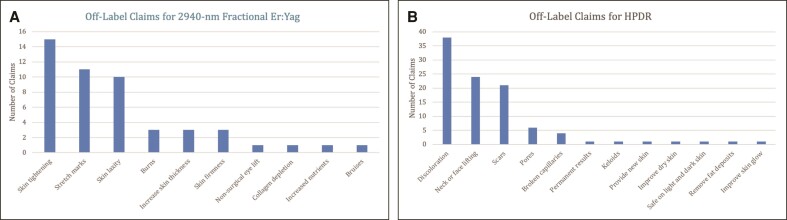
(A) Off-label claims for 2940 nm fractional erbium-doped yttrium aluminum garnet. (B) Off-label claims for helium plasma dermal resurfacing (HPDR).

More than half of all websites advertised an off-label claim (*n* = 103; 51.5%), with 38 making 2 or more off-label claims (19%; Table 1). Forty-nine off-label claims were identified for 2940 nm fractional Er:YAG, including skin tightening (*n* = 15), treatment of stretch marks (*n* = 11), improvement in skin laxity (*n* = 10), treatment of burn scars (*n* = 3), and increased skin thickness (*n* = 3; Figure 2A). One hundred off-label claims were identified for HPDR, including treatment of discoloration (*n* = 38), neck or face lifting (*n* = 24), treatment of scars (*n* = 21), pores (*n* = 6), and broken capillaries (*n* = 4; Figure 2B). Furthermore, only 9 sites mentioned a Fitzpatrick limitation for helium lasers when used for dermal resurfacing.

Both plastic surgeon and nonplastic surgeon-run websites made at least 1 off-label claim (54.7% and 48.6% of sites, respectively), with no statistically significant difference between the 2 (*P* = .384; Table 2). When broken down by provider subtype (surgeons, nonsurgeons, dermatologists, or nonphysician providers), dermatologists were most likely to advertise off-label uses (82.4%), whereas nonsurgeons had the lowest rate of off-label advertising (33.3%; *P* = .012; Table 3). Websites advertising 2490 nm fractional Er:YAG were more likely to make off-label claims compared with HPDR (61% vs 46%; *P* = .033; Table 4).

**Table 2.** Off-label Claims by Plastic Surgeon vs Nonplastic Surgeon

	Nonplastic surgeon site (*n* = 105)	Plastic surgeon site (*n* = 95)	Total (*n* = 200)	*P*-value
Any off-label claim				.384^a^
	51 (48.6%)	52 (54.7%)	103 (51.5%)	
>1 off-label claim				.732^a^
	19 (18.1%)	19 (20.0%)	38 (19.0%)	
Total off-label claims				.678^b^
0	51 (48.6%)	52 (54.7%)	103 (51.5%)	
1	34 (33.3%)	24 (25.3%)	59 (29.5%)	
2	14 (13.3%)	14 (14.7%)	28 (14.0%)	
3	2 (1.9%)	4 (4.2%)	6 (3.0%)	
4	3 (2.9%)	1 (1.1%)	4 (2.0%)	
Laser type				<.001^a^
2940 nm fractional Er:YAG	65 (61.9%)	35 (36.8%)	100 (50.0%)	
Helium plasma	40 (38.1%)	60 (63.2%)	100 (50.0%)	

Er:YAG, erbium-doped yttrium aluminum garnet. ^a^Pearson's χ^2^ test. ^b^Trend test for ordinal variables.

**Table 3.** Off-label Claims by Provider Subtype

	Surgeon (*n* = 136)	Nonsurgeon (*n* = 33)	Dermatologist (*n* = 17)	Other (*n* = 14)	*P*-value
Any off-label claim					.012^a^
	71 (52.2%)	11 (33.3%)	14 (82.4%)	7 (50.0%)	
>1 off-label claim					.083^a^
	24 (17.6%)	11 (33.3%)	1 (5.9%)	2 (14.3%)	
Total off-label claims					.004^b^
0	71 (52.2%)	11 (33.3%)	14 (82.4%)	7 (50.0%)	
1	41 (30.1%)	11 (33.3%)	2 (11.8%)	5 (35.7%)	
2	18 (13.2%)	7 (21.2%)	1 (5.9%)	2 (14.3%)	
3	5 (3.7%)	1 (3.0%)	0 (0.0%)	0 (0.0%)	
4	1 (0.7%)	3 (9.1%)	0 (0.0%)	0 (0.0%)	
Laser type					.003^a^
2940 nm fractional Er:YAG	56 (41.2%)	21 (63.6%)	12 (70.6%)	11 (78.6%)	
Helium plasma	80 (58.8%)	12 (36.4%)	5 (29.4%)	3 (21.4%)	

Er:YAG, erbium-doped yttrium aluminum garnet. ^a^Pearson's χ^2^ test. ^b^Trend test for ordinal variables.

**Table 4.** Off-label Claims by Laser Type

	2940 nm fractional Er:YAG (*n* = 100)	Helium plasma (*n* = 100)	*P*-value
Any off-label claim			.034^a^
	59 (59.0%)	44 (44.0%)	
>1 off-label claim			<.001^a^
Yes	7 (7.0%)	31 (31.0%)	
No. of off-label claims			<.001^b^
0	59 (59.0%)	44 (44.0%)	
1	34 (34.0%)	25 (25.0%)	
2	6 (6.0%)	22 (22.0%)	
3	1 (1.0%)	5 (5.0%)	
4	0 (0.0%)	4 (4.0%)	

Er:YAG, erbium-doped yttrium aluminum garnet. ^a^Pearson's χ^2^ test. ^b^Trend test for ordinal variables.

## DISCUSSION

Cosmetic lasers have become an integral part of the practice of aesthetic medicine. First approved by the US FDA in 1989 for permanent removal of pigmented hair, the technology and applications of lasers have evolved significantly.^17^ Since 2012, the use of energy-based devices by dermatologic surgeons has increased by 106%, reaching 4.1 million total procedures in 2019.^18^ Concurrently, patients are increasingly turning to commercial websites and publicly available search engines to find health-related information.^19^ However, research demonstrates that online information on nonsurgical aesthetic procedures is often of poor quality and dominated by commercial entities, thus potentially misinforming patients.^20^ Medical practitioners may be unaware of FDA-advertising regulations, unintentionally marketing off-label claims.

The results of our study demonstrated a significant discrepancy between FDA-approved indications of use and off-label advertisements made by medical providers. Despite regulations prohibiting off-label marketing of medical devices, we found that more than half (51.5%) of online practices made at least 1 off-label advertisement about a cosmetic laser and 19% made 2 or more off-label claims. We elected to analyze dermatologists and plastic surgeons separately from other surgical specialists as many professionals in these fields regularly receive dedicated cosmetic laser training in residency.^21^ Our analysis illustrates that plastic surgeons were just as likely to make off-label claims as their nonsurgeon counterparts (*P* = .384). However, when broken down by specialty, dermatologists displayed a higher rate of off-label claims, whereas other nonsurgical specialists had the lowest rate (82.4% and 33.3%, respectively; *P* = .003).

The 2 main routes for medical device approval are the premarket approval (PMA) application, which requires clinical trials, and the 510(k) premarket notification, which exempts medical devices from clinical trials if they are deemed substantially equivalent to an existing device. The goal of the 510(k) regulatory process is to minimize the burden and expense of bringing new devices to the market, while ensuring their safety and effectiveness. This process is significantly faster, less costly to the manufacturer, and requires less premarket testing than the PMA; therefore, it is not uncommon for most cosmetic lasers and radiofrequency devices to be approved through the 510(k) pathway. In fact, the 510(k) pathway is responsible for >99% of device reviews and ∼3000 annual approvals.^22^ Alternatively, <20 devices are approved annually through the more rigorous PMA application.^22^ To meet the burden of proof for a 510(k) approval, manufacturers must provide safety and efficacy data for review. In a sample of 50 newly cleared implants, publicly available scientific data were identified for only 8 implants (16%).^23^ Furthermore, existing literature demonstrates a high rate of recall for 510(k) devices, suggesting that some of these devices may be approved with insufficient long-term data.^24^ Given the current regulatory framework, practitioners should refrain from marketing possible off-label uses until further experience and scientific research can assess device safety and effectiveness.

There are certain limitations to this study. While all data were collected at a single time point, we were not able to ascertain when the information posted online was published. The authors attempted to reduce sample bias by disabling location-based search results; however, the authors acknowledge that Google uses a proprietary algorithm that may impact the search results. Nevertheless, this search strategy mimics that of a patient searching for information regarding lasers. There are also inherent limitations to interpreting a device's indications of use listed on the 510(k) approval. The 510(k) approval only reflects the indications for which its manufacturers have sought approval. These may be delayed in comparison with current research, demonstrating new capabilities of a device. For example, Radiesse (calcium hydroxyapatite; Bioform Medical Inc., San Mateo, CA) was initially approved for use in bone augmentation. Despite this, physicians commonly used it “off-label” for cosmetic purposes until it received formal approval in 2006 for the correction of facial lipoatrophy and the reduction of moderate-to-severe facial wrinkles.^25^ The 2940 nm fractional Er:YAG and HDPR lasers examined in our study received their latest 510(k) notification update in 2018 and 2023, respectively. Numerous case series and clinical trials have been undertaken since, which are not reflected in the 510(k) indications of use. For instance, several clinical trials examining HPDR have demonstrated its potential for treatment of brown spots and enlarged pores, although these indications are not currently listed in the 510(k) and therefore, still considered off-label uses.^26^ It is unknown whether manufacturers of these lasers are actively pursuing approval for these and other indications not currently included in our study. Lastly, we chose to consider any deviation from the approved indication as off-label. For example, while “acne scars” are an approved indication for 2940 nm fractional Er:YAG, we considered “burn scars” to be an off-label indication. Although it is unknown whether regulatory bodies would regard these distinctions as significant, we adopted this approach to more accurately capture the nuances of off-label advertising. Additionally, while patient-directed flyers led the FDA to send a letter prohibiting physicians from advertising off-label uses to the general public, our analysis focuses on online promotion of products, which has now effectively replaced print-based advertising.

Off-label *use* of pharmaceuticals and medical devices remains a common and legal practice, so long as their use is based on “firm scientific rationale and on sound medical evidence”; however, it is illegal to *advertise* for such uses if not FDA approved.^27^ Because patients continue requesting treatments based on direct-to-consumer marketing, physicians should understand the risks and benefits of each medical device they employ and market the devices ethically and legally. Otherwise, patients may undergo treatments that do not have true clinical effectiveness or result in harm, potentially subjecting practitioners to certain liabilities as a consequence of illegal off-label advertising.^10,28^ Future studies should be undertaken to comprehensively examine the promotion of other devices on the market. Additionally, these studies should examine geographical differences as well as the impact of academic or research involvement by providers on their rates of off-label advertising.

## CONCLUSIONS

Despite FDA regulation of medical devices, online claims regarding the indications and capabilities for popular medical lasers vary widely. Plastic surgeons are just as likely to make off-label claims as their nonsurgeon counterparts. Patients who seek information regarding potential aesthetic laser treatments may encounter inaccurate and differing claims for these treatments, potentially leading to false expectations and poor patient outcomes.

## Data Availability

All data utilized in this study were sourced from publicly available Google searches and are available from the first author upon request.
